# Infectious meningitis and encephalitis: A primer for acute care practitioners

**DOI:** 10.1177/17511437261437982

**Published:** 2026-04-06

**Authors:** Martin Beed, Chit Wong, Rob A Dineen, Louise Berry, Peter G Brindley

**Affiliations:** 1 Nottingham University Hospitals, UK; 2 University of Alberta, Edmonton, AB, Canada

**Keywords:** meningitis, encephalitis, intensive care, neuro intensive care, teamwork

## Abstract

Meningitis and encephalitis affect all ages, are prone to misdiagnosis and outcome can be devastating. We provide this common primer for all in the sepsis “chain-of-survival.” Meningitis equals inflammation/infection of the protective membranes that cover the brain; whereas encephalitis affects the brain parenchyma. Meningitis is more common, but they can co-exist as meningoencephalitis. Encephalitis can also affect the spinal cord (encephalomyelitis). Worldwide, meningitis affects 2.5 million people annually, and kills over 200,000. Central nervous system (CNS) infections account for 3.9% of all UK intensive care unit (ICU) infections, and 0.7% of adult ICU admissions. While this means these are not common causes for admission, they do have high morbidity and mortality. The median ICU stay is 4 days, of which 3 days was the median spent requiring advanced respiratory support or support for more than one organ. The median in-hospital stay is 20 days. Most admissions come through the emergency department (ED). Signs and symptoms can be vague and varied; hence potential misdiagnosis as flu, psychiatric disorders, intoxication, even hangover. The median time between hospital admission and transfer to ICU is 1 day, and by this time approximately one-third are comatose and one-sixth need respiratory support. The risk of misdiagnosis matters given high mortality and morbidity: 18%–25% die in hospital and 1-in-10 survivors lose independence. During the past 20 years mortality has fallen, but those left with some form of permanent disability remains constant at nearly 40%. Fortunately, early recognition and treatment can greatly improve outcome. Regarding diagnosis, history and physical examination still have great value. Next, lumbar puncture (LP) should be expedited unless contraindicated by coagulopathy, skin infection, or raised ICP. LP testing should incorporate opening pressure, microscopy, culture and cell count, glucose and protein and often polymerase chain reaction (PCR) for meningococcus, pneumococcus, herpes simplex virus (HSV1&2), varicella (VZV) and enterovirus. Radiologically, head computed tomography (CT) is first line. It may reduce the risk of LP by excluding pathologies likely to trigger herniation. CT is indicated if their Glasgow Coma Score (GCS) is falling or ⩽9, or if seizures, focal neurological signs or papilloedema. Normal CT cannot rule out raised ICP, but LP is avoided if the CT shows herniation, basal cistern or foramen magnum effacement, cerebral swelling, intracranial lesions/collections with mass effect or obstructive hydrocephalus. Magnetic resonance imaging (MRI) is logistically tougher but better at detecting meningitis/encephalitis. MRI can suggest the causative organisms, along with complications such as infarct, pus and parenchymal changes. Treatment centres on prompt antimicrobials: usually a third-generation intravenous (IV) cephalosporin, typically within 1 h, and at an increased (i.e. “meningitis”) dose. Intravenous amoxicillin is added in the elderly or immunocompromised, plus aciclovir if viral encephalitis is plausible. Treatment delays (over 4 h) are associated with increased mortality. Over half (57%) of patients that require ICU develop intracranial complications, most frequently ischaemia, cerebral oedema and ventriculitis. In short, these diseases are life-threatening but manageable if we do the simple stuff right. . .and right away.

*“Excellence is doing ordinary things extraordinarily well”*

John W Gardner, Former U.S Secretary of Health Education and Welfare

## Introduction

Meningitis and encephalitis affect all ages, are prone to misdiagnosis and the outcome can be devastating. For these reasons, we provide this common primer for all those in the sepsis “chain-of-survival.”^1^

Meningitis is inflammation and infection of the meninges, the protective membranes covering the brain and spinal cord; whereas encephalitis affects the brain parenchyma. Meningitis is more common, but the two can co-exist as meningoencephalitis. Encephalitis can also affect the spinal cord, in which case it is called encephalomyelitis. Worldwide, meningitis affects 2.5 million people annually, and results in over 200,000 deaths.^2^

In the UK, central nervous system (CNS) infections account for 3.9% of all intensive care unit (ICU) infections, and 0.7% of adult intensive care unit admissions.^3,4^ While this means that these are not a common cause for admission, cases do have high morbidity and mortality. The median length of ICU stay is 4 days, of which 3 days was the median time spent requiring advanced respiratory support or support for more than one organ system.^4^ The median length of hospital stay is 20 days.

Most admissions come through the emergency department (ED). Signs and symptoms can be vague and varied and hence these conditions have been misinterpreted for flu, psychiatric disorders, intoxication and even hangover.^5–7^ The median time between hospital admission and transfer to ICU is 1 day, and by this time approximately one-third are comatose and one-sixth need respiratory support.^8^

The risk of misdiagnosis matters given high mortality and morbidity – 18%–25% die in hospital and 1-in-10 survivors lose their independence. Over the past 20 years there is evidence that mortality has fallen, but those left with some form of permanent disability remains constant at nearly 40%.^4^ Fortunately, early recognition and treatment can greatly improve outcome.

Regarding diagnosis, history and physical examination still have great value. Next, lumbar puncture (LP) should be expedited unless contraindicated by coagulopathy, skin infection at the needle site, or raised ICP (more details below). LP testing should incorporate opening pressure, microscopy, culture and cell count, glucose and protein and often polymerase chain reaction (PCR) for meningococcus, pneumococcus, herpes simplex virus (HSV1&2), varicella (VZV) and enterovirus.

Radiologically, head computed tomography (CT) is first line. It may reduce the risk of an LP by excluding pathologies likely to trigger herniation. CT is indicated if their Glasgow Coma Score (GCS) is falling or ⩽9, or if there are seizures, focal neurological signs or papilloedema.^9,10^ A normal CT cannot rule out raised ICP, but an LP is avoided if the CT shows herniation, basal cistern or foramen magnum effacement, cerebral swelling, intracranial lesions/collections with mass effect or obstructive hydrocephalus.^11,12^ Magnetic resonance imaging (MRI) is logistically tougher but better at detecting meningitis/encephalitis. MRI can also suggest the causative organisms, along with complications such as infarct, pus and parenchymal changes.

Treatment centres on prompt antimicrobials. This usually means a third-generation intravenous (IV) cephalosporin, typically within 1 h and administered at an increased (i.e. “meningitic”) dose. Intravenous amoxicillin is added in the elderly or immunocompromised, plus aciclovir if viral encephalitis is plausible.^9,10^ Treatment delays (over 4 h) are associated with increased mortality.^13^ Over half (57%) of patients that require ICU develop intracranial complications, most frequently ischaemia, cerebral oedema and ventriculitis.^14^ In short, these diseases are life-threatening but manageable if we are vigilant, work as a team, and do the simple stuff right. . .and right away.

## Causes of infectious meningitis and encephalitis

As outlined in Tables 1 and 2, infectious meningitis and encephalitis are caused by a wide range of bacteria, mycobacteria, viruses, fungi, parasites and spirochetes. *Streptococcus pneumoniae* is the culprit in approximately 60% of bacterial cases. *Neisseria meningitidis* is the perpetrator in approximately 7%, and *Listeria monocytogenes* in 5.5% (with a preference for the old or immunocompromised).^14^ *Herpes simplex* viruses 1 (HSV1), *Varicella zoster* (VZV) and HSV2 account for 32%, 15% and 4% of all viral encephalitis, respectively.^8,13^ In immunocompromised states, less common micro-organisms occur more frequently. For example, the most common cause in HIV patients is the fungus Cryptococcus.^15^

**Table 1.** CNS infections and adult intensive care unit admissions.

Donovan et al. 2024.^4^	Sonneville et al. 2023.^8^
• Meningitis unspecified (26.6%). • Bacterial meningitis non-meningococcal (15.4%). • Meningococcal meningitis (5.4%). • Viral meningitis (1.8%). • Tuberculous meningitis (0.7%). • Encephalitis (33.3%). • Intracranial abscess (13.5%). • Infected CSF shunt (3.3%).	• Acute bacterial meningitis (41.8%). • Infectious encephalitis (23.7%), of which: ○ Viral causes (17.1%). ○ Subacute bacterial causes (4.2%). ○ Fungal/parasitic causes (2.4%).

**Table 2.** Pathogens associated with infective meningitis/encephalitis.

Bacteria.	• Streptococcus pneumonia. • Neisseria meningitides. • Group B streptococci. • Haemophilus influenzae. • Listeria monocytogenes (in the elderly, pregnant, those affected by alcohol misuse, or immunocompromise). • Leptospirosis (associated with travel, gardening, or outdoor water sports). • Mycobacterium tuberculosis (associated with HIV). • *Treponema pallidum* (syphilis) • *Rickettsial* infections (e.g. Rocky Mountain or Mediterranean Spotted fevers).
Bacteria associated with surgery, trauma or head/neck infection (sinusitis).	• Staphylococcus aureus. • Pseudomonas. • Haemophilus influenzae.
Viruses.	• Herpes simplex virus 1 and 2. • Enterovirus. • Varicella zoster. • Measles. • Mumps. • HIV. • Influenza. • Lymphocytic choriomeningitis (LCMV). • SARS-CoV2/COVID19.
Arthropod borne viruses (arboviruses).	• West Nile. • Japanese encephalitis. • Rift valley. • Yellow fever. • Tick borne encephalitis. • Dengue (rare complication).
Tick borne bacteria.	• *Borrelia* (Lyme disease)
Fungal (associated with immunocompromise and HIV infection).	• Cryptococcus neoformans/cryptococcus gattii. • Coccidiodes. • Blastomyces. • Candida. • Histoplasma. • Aspergillus.
Parasites.	• Toxoplasma gondii (associated with HIV). • Malaria • Schistosoma. • Taenia solium (cysticercosis). • Toxicariasis. • Angiostrongylus.

Direct infection and contiguous spread can occur following acute or chronic sinusitis, CNS surgery or trauma where the dura has been breached.^9^ In rare cases infection has been caused by epidural or intrathecal injections.^16,17^ Bloodstream infections can spread to the brain, and cardiac infections (namely endocarditis) can embolise to the central vessels as “septic emboli” which can cause ischaemia. Some septic emboli develop into mycotic aneurysms, where the infected blood vessel wall becomes dilated (the term mycotic fallaciously suggests the cause is always fungal). There is also a variation of *Streptococcus pneumoniae* meningitis known as Osler’s triad because it consists of meningitis, endocarditis and pneumonia (it is also called Austrian syndrome – named after Robert Austrian, rather than the country).^18^

Encephalopathy and neuro-inflammation also occur in the absence of direct CNS involvement. Examples include malaria, influenza and severe acute respiratory syndrome coronavirus 2 (SARS-CoV2).^19^

## Differential diagnosis and aseptic meningitis

Patients with can present with non-specific signs and symptoms including altered consciousness, headache, seizure, behavioural change and focal neurology signs. The differential for meningitis and/or encephalitis is broad and ranges from intoxication, through haemorrhagic or thrombotic stroke and onto solid tumours (including metastases), haematologic malignancies (lymphoma or leukaemia), autoimmune encephalitis and vasculitis. This means we need a high index of suspicion while accepting that there are many impersonators.

Simply put, if a condition is associated with inflamed meninges we use the term meningitis, even if the cause is not clear.

The term aseptic meningitis refers to inflammation of the meninges that is either not bacterial in origin, or caused by bacteria that cannot be grown in standard culture medium. It can be broadly divided into infectious and non-infectious causes. Approximately half are not precisely diagnosed, in which case they are called idiopathic. The most common cause of aseptic meningitis is a virus – especially enteroviruses (i.e. Coxsackie and Echovirus). It can also be fungal (i.e. Cryptococcus and Coccidioides) or from a parasitic (malarial or cysticercosis), mycobacterium, or spirochete (Lyme or syphilis).^20^

Non-infectious causes of aseptic meningitis include subarachnoid haemorrhage (SAH; due to blood products irritating the meninges), migraine (likely due to irritation of the trigeminal nerve), and migraine-like headaches such as HaNDL (syndrome of transient Headache and Neurological Deficits with cerebrospinal fluid Lymphocytosis).^21^ Aseptic meningitis can also be associated with connective tissue disorders and vasculitis such as systemic lupus erythematosus and Behcet’s, Sjogren’s, and Kawasaki’s, and granulomatous diseases such as sarcoidosis.

Carcinomatous meningitis is caused by direct invasion of the meninges by cancer cells, thereby leading to a meningo-encephalitic picture. Paraneoplastic syndromes are a separate entity where neurological syndromes are associated with specific antibodies, some of which cause an encephalitic presentation and CSF lymphocytosis. Paraneoplastic syndromes typically precede the onset of the malignancy, whereas carcinomatous is a late, usually terminal event in advanced cancer.

Drug-induced aseptic meningitis can be triggered by non-steroidal anti-inflammatory drugs, antibiotics (sulphonamides, penicillins) and intravenous immunoglobulin. Aseptic meningitis can also occur, albeit rarely, after vaccines notably measles, mumps, rubella (MMR), yellow fever, rabies, pertussis and even influenza.^20^

A number of patients who end up requiring ICU will turn out to have a diagnosis of autoimmune encephalitis, most commonly anti-NMDA receptor disease. Whilst there is an overlap in ICU presentation (e.g. altered behaviour, delirium, decreased consciousness and seizures), the behavioural element sometimes predominates to such an extent that these patients can present via psychiatry with delusions, hallucinations or catatonia. To further complicate things, prior HSV encephalitis is well associated with anti-NMDA receptor encephalitis.^22^

## Clinical presentation

Presentation varies. This is especially true in elderly or immunocompromised patients, namely those that do not mount a substantial inflammatory response. Signs and symptoms can be as subtle as a low-grade fever or flu-like prodrome.

These can be accompanied by meningismus (neck stiffness, headache and photophobia), behavioural changes and altered consciousness or minor personality changes (Table 3).^23^ Obviously the vast majority of headaches are not meningitis, but vigilance is key.

**Table 3.** Signs and symptoms associated with infectious meningitis and encephalitis.

Signs and symptoms associated with infection.		
Infection^a^	• Pyrexia. • Tachycardia. • Tachypnoea. • Rigours. • Raised white cell count. • Raised inflammatory markers (e.g. C-reactive protein).	
Sepsis	• Hypotension. • Cardiovascular collapse. • Disseminated intravascular coagulation. • Acute respiratory distress syndrome.	
Rash	• Purpuric, non-blanching (meningococcal rash). • Vesicular (viral meningitis/encephalitis).	
Neurological signs and symptoms.		
Meningism	• Headache and/or neck stiffness. ○ Positive Kernig’s or Brudzinski’s sign. • Photophobia. • Nausea and vomiting. • Papilloedema.	
Altered neurology	• Abnormal behaviour. • Seizures. • Focal neurological signs such as cranial nerve palsies. • Decreased consciousness. ○ Signs associated with decreased consciousness, e.g. loss of airway reflexes.	
Frequency of selected signs and symptoms^9,10,23–28^		
	Bacterial meningitis (%)^b^	Viral meningoencephalitis (%)^c^
Pyrexia	77–97	72
Rash	20–52	Not reported
Headache	58–87	88
Neck stiffness	69–83	36
Nausea and vomiting	45–74	54
Altered mental status	30–69	68

a Bradycardia and bradypnoea may occur as a result of neurological complications. Hypothermia and neutropaenia may be present, particularly at extremes of age or with immune compromise.

b The ranges reported cover the results from five studies from different countries.^24–28^

c Within the study by Schwitter et al. 32% of cases contributing to the findings are tick-borne encephalitis infections.^23^

Differentiating between meningitis and encephalitis may also be difficult. The diagnosis of encephalitis requires altered mental status (decreased consciousness, lethargy, or personality change), combined with any two of: fever, general or focal seizures, new focal neurology, raised CSF white cell count, EEG or neuroimaging suggestive of encephalitis.^29^

Physical exam should focus on temperature, blood pressure and heart rate, plus tests for meningism (once again, this means neck stiffness, headache and photophobia). These tests include Kernig’s sign (passive knee extension with the hip flexed causes head/neck pain) and Brudzinski’s sign (neck flexion triggers hip and knee flexion). These tests have high specificity, but low sensitivity.^8^ There means that if absent the patient could still have meningitis. There is also the Jolt Accentuation of Headache (JAM) test, where the baseline headache worsens with several quick horizontal head rotations. This test has moderate sensitivity and specificity but is uncomfortable and rarely used.^30^ It is also potentially dangerous in cases of vertebral dissection.

Meningitis can be associated with raised intracranial pressure (ICP) resulting in papilloedema, namely optic disc swelling seen on fundoscopy. As such, clinicians should maintain basic fundoscopic skills. Handheld retinal cameras may also be increasingly used. In young infants, a bulging fontanelle can indicate raised ICP.

*Neisseria meningitides* infections are accompanied by septicaemia in 70% of cases.^9,10^ Meningococcal septicaemia is characterised by septic shock with a spreading, non-blanching, purpuric rash that becomes confluent and can be associated with multisystem organ failure. Importantly, this “haemodynamic” presentation of *Neisseria meningitides* is different from the “neurological” presentation of meningitis and the two rarely co-exist.^31^ Viral encephalitis/meningitis is sometimes associated with a vesicular rash, such as varicella blisters.

Meningitis and encephalitis occur across all ages, while the mean age of those requiring ICU admission is 54, with 55% male predominance.^4^ Age can be a clue as to the causative agent, with older patients more at risk from *Listeria monocytogenes*, whilst neonates are susceptible to HSV, acquired during vaginal delivery. Infants are more susceptible to *Haemophilus influenza B* (HiB) and measles, and we should expect to see more given increased vaccine hesitancy.^32^

Those newly exposed to close-contact groupings – for example new college students – are at increased risk from *Neisseria meningitidis.* Clusters of *meningococcal* infections also follow exposure from pilgrimages such as Hajj, and *meningococcal* epidemics occur in the “meningitis belt”: an area of sub-Saharan Africa from the west coast across to Ethiopia.^2,33^

Foreign travel is associated with viral infections that can cause encephalitis, particularly arboviruses. However, many viruses are increasingly found outside their traditional range. For example, West Nile virus arrived in North America in 1999 and the UK in 2025, whilst tickborne encephalitis has been in the UK since 2019.^34–36^ With the exception of toxoplasmosis, parasitic infections are also strongly associated with foreign residency and travel. Worldwide each year, over half-a-million people get malarial meningitis. This is caused by the plasmodium falciparum parasite and should be suspected in those from endemic area such as sub-Saharan Africa, and especially in children under 5 years. Of note, malarial meningitis can coexist with bacterial meningitis, or other rare eosinophilic meningitides such as those associated with tapeworms or amoebas.

Immunosuppression changes the likely pathogen. For example, human immunodeficiency virus (HIV) and prior chemotherapy are associated with Tuberculosis (TB), fungi, and toxoplasmosis.^13,16^

## Lumbar puncture

Lumbar puncture (LP) is the most effective test to identify infectious meningitis/encephalitis. Accordingly, skills need to be kept up. We have therefore enclosed a link that offers an anatomic review and practical tips and would recommend considering the purchase of task trainers and simulators.^37^ An LP should be performed unless there are contra-indications. As above, these include raised ICP, coagulopathy (platelets <50 × 10^9^/L or INR > 1.3), or skin damage/infection at the needle insertion site.

Computed tomography (CT) of the brain is performed before an LP to look for features associated with raised ICP (and in particular exclude a mass lesion, especially if obstructing the flow of CSF) and is recommended if the Glasgow Coma Score (GCS) is nine or less (or progressively falling) or if there are focal neurological signs, multiple seizures or papilloedema.^9,10^ This LP should ideally be performed within an hour of presentation, along with blood cultures and throat swabs for meningococcal culture and viral pathogen PCR (e.g. influenza, SARS-CoV2). Table 4 outlines how cerebrospinal fluid (CSF) tests can be divided into first-line (red boxes), second-line (amber boxes), as well as outlining other tests that are available (the latter includes antibody tests for autoimmune diseases including encephalitis).^9,10,38,39^ A “paired” blood glucose should also be taken to compare the CSF/blood glucose ratio. Second-line tests, for example for fungal or TB, are guided by microbiologists, whilst autoimmune disease tests may be guided by neurologists.^22,38^ TB meningitis is paucibacterial, meaning CSF culture requires more volume than for other tests.

**Table 4.** Lumbar puncture cerebrospinal fluid (CSF) tests and findings.

Parameter	Volume required	Normal range	Findings with the following diseases				
			Bacterial	Viral	Tuberculosis	Fungal	Autoimmune
**Opening pressure**	**Microscopy and culture** 1–3 mL	10–20 cmH_2_O	Raised	Normal or raised	Raised	Normal or raised	Normal or raised
**Appearance and white cell count**	TB culture/PCR 7 mL	Clear 0–5 cells/μL (lymphocytes)	Cloudy >1000 cells/μL (neutrophils)	Clear 5–1000 cells/μL (lymphocytes)	Cobweb-like 10–500 cells/μL (lymphocytes)	Clear or cloudy 0–500 cells/μL (lymphocytes)	Clear 5–10 cells/μL (lymphocytes)
***Protein***	1.5 mL	0.15–0.45 g/L	>1 g/L	0.4 – 1 g/L	1–5 g/L	0.4–2.5 g/L	0.45–1 g/L
***Glucose*** (CSF:serum ratio)	1 mL	>60%	<40%	Usually normal	<30%	<40%	>60%
Lactate	1 ml	<2 mmol/L	>2 mmol/L	<2 mmol/L	>2 mmol/L	>2 mmol/L	<2 mmol/L
PCR tests	**Meningococcus/** **pneumococcus** 1 mL.	**–**	Meningococcus, Pneumococcus, E. coli, H. influenzae, Listeria, GBS	HSV1, HSV2, VZV, Enterovirus, Parechovirus, HPV6, CMV	TB PCR	Cryptococcus neoformans, Cryptococcus gattii	**–**
	**HSV1/2, VZV, Enterovirus** 1.5 mL						
	16sRNA 1 mL						
Antibodies and antigens	CrAg 0.2 mL.	**–**	**–**	**–**	**–**	CrAg	AMPAR, CASPR2, DPPX, GABAAR/BR, GAD, GlyR, IgLON5, LGI1, NMDAR,
	Other antibody tests (variable volume).						

Tests listed in bold within red boxes are first-line tests. Tests in amber boxes are second line (see accompanying text on microbiological investigations).

16sRNA: ribonucleic acid from 16s unit of bacterial ribosome; AMPAR: α-amino-3-hydroxy-5-methyl-4-isoxazolepropionic acid receptor; CASPR2:contactin-associated protein-like 2; CMV: cytomegalovirus; CrAg: cryptococcus antigen test; CSF: cerebrospinal fluid; DPPX: dipeptidyl-peptidase-like protein 6; GABA(AR or BR): gamma-aminobutyric acid A/B receptor; GAD: glutamic acid decarboxylase; GBS: group-B streptococcus; GlyR: glycine receptor; HPV6: human papilloma virus 6; HSV1/2: herpes simplex virus; IgLON5: immunoglobulin-like cell adhesion molecule 5; LGI1: leucine-rich glioma inactivated; NMDAR: N-methyl-D-aspartate receptor; PCR: polymerase chain reaction; TB: tuberculosis; VZV: varicella zoster virus.

Other tests may also be indicated, for example: malaria (via thick and thin blood smears and rapid diagnostic testing) if there has been travel to an endemic area, or a throat swab and stool sample for enterovirus in immunocompromised patients. Where a rash is present, skin scrapings or pustule aspirates can be sent. HIV and syphilis testing should be considered, and serum/CSF cryptococcus antigen (CrAg) testing may be necessary for HIV positive patients. These authors were taught to draw an extra tube and have the lab save it. This is in case additional tests are asked for later. It was known as a “did-you tube,” as in “did you also send for cryptococcal antigen?” The differential diagnoses for when no causative organism can be found are listed in Table 5.

**Table 5.** Potential causes of aseptic meningitis (namely, where no causative organism is found on initial lumbar puncture samples).

• Partially treated bacterial meningitis (e.g. LP delayed after antibiotics given). • Viral meningitis (e.g. HSV 1/2, VZV). • Bacterial meningitis due to TB, syphilis, or rickettsial infections. • Fungal meningitis (e.g. *Candida*, *Cryptococcus*, *Histoplasma*, *Coccidioides*). • Protozoal meningitis (e.g. cerebral malaria, or Toxoplasma). • Lymphoma. • Vasculitis. • Subarachnoid haemorrhage.

## Radiological investigations

Head CT is the first line neuroimaging modality. This is because it is fast and relatively availability (certainly compared to MRI). This, in turn, minimises treatment delays. CT has low sensitivity but can be helpful for identifying sources of infection, detecting and monitoring complications and if neurosurgical drainage is indicated.

Importantly, the CT is often normal in both meningitis and encephalitis. This means it is better understood as a screen for complications and other diagnoses, rather than as a diagnostic tool for meningoencephalitis. However, there can be pus in CSF spaces, most often the occipital horns. In encephalitis, parenchymal oedema can produce reduced attenuation with or without swelling. More rarely, there can be haemorrhage or even overt tissue destruction and cavitation. CT can also identify intracranial infection eroding into the intracranial compartment from paranasal sinuses or petrous temporal bones. There can also be parenchymal abscesses, extra-axial collections and hydrocephalus, plus areas of infarction with bacterial meningitis. A contrast CT may also identify leptomeningeal enhancement and secondary venous thrombosis.

LP is avoided if there are CT findings that portend a greater risk of brain herniation. These include existing herniation, effacement of basal cisterns or foramen magnum, cerebral swelling, intracranial lesion/collection with mass effect and obstructive hydrocephalus. Unfortunately, a normal CT cannot entirely exclude raised ICP, hence an LP can still trigger herniation, albeit rarely.^11,12^

MRI is less readily available than CT. It also takes longer and comes with the concerns of placing a potentially agitated patient in a tight tube, and without easy access if they deteriorate. However, it does have greater sensitivity for characterising meningitis and encephalitis. This is because it can identify subtle parenchymal involvement. Contrast enhancement can also highlight leptomeningeal and ependymal inflammation, cranial nerve and inner ear involvement and small abscesses and infarcts.

Anatomic changes can strongly suggest specific pathogens, particularly in the context of opportunistic infections. For example, HSV and parechovirus have characteristic parenchymal distributions, whilst basal meningitis is more characteristic of TB, as are perforator territory abscesses in cryptococcal meningitis.

Diffusion-weighted imaging (DWI) MRI is exquisitely sensitive for intracranial pus and able to detect small abscesses and collections, and distinguish them from aseptic effusions. It is also sensitive to parenchymal ischaemia and other cytotoxic processes. This means it can demonstrate meningitis-related infarcts and cytotoxic oedema in encephalitis.

Cranial ultrasound requires the requisite expertise, but can detect and then monitor the complications of meningitis in infants, particularly neonates. Pertinent changes include parenchymal collections, hydrocephalus and venous thrombosis.

## Electroencephalography (EEG)

EEG is not a key test in infectious meningitis/encephalitis because there are no diagnostic EEG changes. The notable exception is HSV encephalitis where non-specific diffuse high amplitude slow waves may be seen, sometimes with temporal lobe spike-and-wave activity, and sometimes periodic lateralised epileptiform discharges (PLEDs).^12^ However, these changes are not specific for HSV and can be seen in other parenchymal pathologies. Despite the relative lack of utility, in one study EEG was found to have been performed in 404/599 cases.^8^ As in other CNS conditions, the role of targeted EEG is to screen for seizures and should focus on those patients with witnessed convulsions, or concerns of non-convulsive epilepsy (namely altered consciousness without an alternate diagnosis), or where there are hard-to-interpret motor findings.^10,13,40^ If there are refractory seizures then continuous EEG may be used to titrate anti-epileptics and achieve burst suppression.

## Therapeutic management

Antibiotics should be given early, usually within an hour, and usually at an elevated dose – so that they penetrate the CNS.^10,13^ Although not as simple as “every minute counts,” there is evidence that underdosing or delaying more than 4 h increases mortality.^41^ This means that antimicrobials may be given prior to LP, even if this decreases diagnostic precision. In these cases, microbiological PCR can be performed later, and inferences made from positive blood cultures.

We need empiric intravenous (IV) antibiotics that penetrate the blood brain barrier (BBB) and reach predictable levels in the CSF. This means our go-to is a third generation cephalosporins (e.g. ceftriaxone or cefotaxime) and at higher doses than for pneumonia (namely 2 g 12-hourly or 2 g 6-hourly respectively). These recommendations come from the UK joint specialities guideline, to which the UK Intensive Care Society (ICS) contributes.^9^ Where penicillin-resistant *S. pneumoniae* is common, vancomycin or rifampicin is added. Intravenous amoxicillin (2 g 4-hourly) is included to target *Listeria monocytogenes* in patients who are immunosuppressed, pregnant, affected by alcohol misuse, diabetic or older (UK guidelines suggest ⩾ 60 years; other guidelines suggest ⩾50).^9,10^ Meropenem is an alternative in penicillin-allergic patients as it covers typical organisms, plus *Listeria*.^9,10^ Piperacillin can cross the BBB into the CSF but penetration is unpredictable or low, unless there is marked meningeal inflammation. It’s partner, tazobactam has even lower penetration and hence is usually inadequate to protect against beta-lactamases producing infections. Accordingly, this otherwise stalwart ICU antibiotic is avoided in bacterial meningitis.

Antiviral treatment of HSV or VZV with IV aciclovir (10 mg/kg 8-hourly) needs to be commenced within 6 h if there are concerns of HSV encephalitis: based on history/examination (decreased consciousness, lethargy, or personality change fever, general or focal seizures, new focal neurology – especially if any of these are rapidly deteriorating), CSF lymphocytosis or neuro-imaging.^13,29^ This is because aciclovir can reduce mortality HSV from >70% to <30%, but needs to be given early.^13^ If in doubt give 72 h of aciclovir and expedite CSF HSV PCR.

There is no evidence for aciclovir treatment of herpes *meningitis* (which can have features of headache and meningism), without associated features of encephalitis/encephalopathy.^9^

Intravenous corticosteroids are recommended for meningitis if they can be given within 12 h of antibiotics being started (and ideally concurrently with the first antibiotic dose). UK joint specialist guidance is that they should then be stopped for all infections other than for *S. pneumoniae* bacterial meningitis (other guidelines also include *H. influenza* as an indication to continue). It is possible their use is associated with decreased mortality and/or reduces the risk of subsequent hearing-loss or neurological sequelae.^8,38^

Dexamethasone has increased penetration and anti-inflammatory potency compared to hydrocortisone, and is usually prescribed 10 mg, every 6-h for 4 days. It is also used in tuberculosis meningitis due to marked swelling in that condition. Routine steroids are not recommended in countries with high HIV rates and where presentations are late.^9,10^ Corticosteroids are also not recommended for viral encephalitis.^13^ Previous case-series evidence did favour dexamethasone in HSV encephalitis, but the subsequent (albeit underpowered) GACHE trial (namely the German trial on Aciclovir and Corticosteroids in Herpes-simplex-virus-Encephalitis) did not.^13,42,43^

Routine prophylaxis with anti-epileptic drugs is not recommended. If seizures occur then they are treated with typical drugs and doses.^16,44^ Routine ICP measurement is not recommended, and, if raised ICP is detected, it is not clear that it benefits from treatment.^9,45^ This is because the effects of mannitol or hypertonic saline are unclear, whilst glycerol, the only osmotic agent to have been trialled, was associated with either no benefit or increased mortality.^9,46^ Inducing hypothermia has been associated with increased mortality in infectious meningitis.^9,47^ Treating pyrexia using Paracetamol/Tylenol is associated with neither harm nor benefit.^10^

## Complications and outcomes

In ICU patients with pneumococcal meningitis over half (57%) develop at least one intracranial complication, including ischaemic lesions, diffuse cerebral oedema and ventriculitis.^14^

Signs of ischaemia and oedema on neuroimaging correlate strongly with poor outcome, and often require prolonged antimicrobials or neurosurgical consult.^14,48^ Even with a normal CT there is a risk of brain herniation, which is more likely in those with respiratory arrest, seizures or declining consciousness.^11,12^

As outlined, infectious meningitis and encephalitis are associated with high mortality and long-term neurological sequelae. Pneumococcal meningitis remains the most lethal, with up to 30% mortality.^14,49^ Sensorineural hearing loss, cognitive impairment, hydrocephalus and seizure disorders are all common, and only about one-fifth of encephalitis survivors able to return to work.^22,43^

In closing, meningitis and encephalitis are common, complex and consequential. They can result in death, disability and legal consequences.^50^ Fortunately, basic knowledge, conscientiousness, and strong teamwork can be literally life-saving.

## References

[1] Hidalgo JL Kumar VK Akech SO , et al. The sepsis chain of survival: a comprehensive framework for improving sepsis outcomes. Crit Care Med 2025; 53(10): e1886–e1892. 10.1097/CCM.0000000000006796 PMC12490334 40668160

[2] Wunrow HY Bender RG Vongpradith A. Global, regional, and national burden of meningitis and its aetiologies, 1990-2019: a systematic analysis for the Global Burden of Disease Study 2019. Lancet Neurol 2023; 22: 685–711. 37479374 10.1016/S1474-4422(23)00195-3 PMC10356620

[3] Vincent J-L Sakr Y Singer M , et al. Prevalence and outcomes of infection among patients in intensive care units in 2017. JAMA 2020; 323: 1478–1487. 32207816 10.1001/jama.2020.2717 PMC7093816

[4] Donovan J Glover A Gregson J , et al. A retrospective analysis of 20,178 adult neurological infection admissions to United Kingdom critical care units from 2001 to 2020. BMC Infect Dis 2024; 24: 132. 38273223 10.1186/s12879-024-08976-z PMC10809450

[5] Latitude: Mum’s meningitis warning 10 years after son dies. BBC News (Online), https://www.bbc.com/news/articles/c87r4d1vn4jo (2024, accessed 11 September 2025).

[6] Bristol woman shares mum’s encephalitis battle to warn others. BBC News (Online), https://www.bbc.com/news/articles/cqx0913njyzo (2025, accessed 11 September 2025).

[7] Encephalitis: ‘I couldn’t remember my boyfriend’. BBC News (Online), https://www.bbc.com/news/newsbeat-51584226 (2020, accessed 11 September 2025).

[8] Sonneville R de Montmollin E Contou D , et al. Clinical features, etiologies, and outcomes in adult patients with meningoencephalitis requiring intensive care (EURECA): an international prospective multicenter cohort study. Intensive Care Med 2023; 49: 517–529. 37022378 10.1007/s00134-023-07032-9

[9] McGill F Heyderman RS Michael BD , et al. The UK joint specialist societies guideline on the diagnosis and management of acute meningitis and meningococcal sepsis in immunocompetent adults. J Infect 2016; 72: 405–438. 26845731 10.1016/j.jinf.2016.01.007

[10] Van De Beek D Cabellos C Dzupova O , et al. ESCMID guideline: diagnosis and treatment of acute bacterial meningitis. Clin Microbiol Infect 2016; 22: S37–S62. 10.1016/j.cmi.2016.01.007 27062097

[11] Joffe AR Martins FDMP Garros D , et al. Lumbar puncture and brain herniation in acute bacterial meningitis: an updated narrative review. J Intensive Care Med 2025; 40(12): 1311–1312. https://journals.sagepub.com/doi/10.1177/08850666251337684 40853372 10.1177/08850666251370334

[12] Fernando SM Tran A Cheng W , et al. Diagnosis of elevated intracranial pressure in critically ill adults: systematic review and meta-analysis. BMJ 2019; 366: l4225. 10.1136/bmj.l4225 PMC6651068 31340932

[13] Solomon T Michael BD Smith PE , et al. Management of suspected viral encephalitis in adults – Association of British Neurologists and British Infection Association National Guidelines. J Infect 2012; 64: 347–373. 22120595 10.1016/j.jinf.2011.11.014

[14] Legouy C Cornic R Razazi K , et al. Intracranial complications in adult patients with severe pneumococcal meningitis: a retrospective multicenter cohort study. Ann Intensive Care 2024; 14: 182. 39699714 10.1186/s13613-024-01405-z PMC11659536

[15] Park BJ Wannemuehler KA Marston BJ , et al. Estimation of the current global burden of cryptococcal meningitis among persons living with HIV/AIDS. AIDS 2009; 23: 525–530. 19182676 10.1097/QAD.0b013e328322ffac

[16] Meyfroidt G Kurtz P Sonneville R. Critical care management of infectious meningitis and encephalitis. Intensive Care Med 2020; 46: 192–201. 31938828 10.1007/s00134-019-05901-w

[17] Centres for Disease Control and Prevention. Bacterial meningitis after intrapartum spinal anesthesia - New York and Ohio, 2008-2009. Centres for Disease Control and Prevention (Online), https://www.cdc.gov/mmwr/preview/mmwrhtml/mm5903a1.htm (2010, accessed 11 September 2025).

[18] Rakočević R Shapouran S Pergament KM. Austrian syndrome - A devastating Osler’s Triad: case report and literature review. Cureus 2019; 11: e4486. 10.7759/cureus.4486 PMC6581326 31259104

[19] Barbosa-Silva MC Lima MN Battaglini D , et al. Infectious disease-associated encephalopathies. Crit Care 2021; 25: 236. 34229735 10.1186/s13054-021-03659-6 PMC8259088

[20] Lee BE Davies HD. Aseptic meningitis. Curr Opin Infect Dis 2007; 20: 272–277. 17471037 10.1097/QCO.0b013e3280ad4672

[21] Fiamingo G Canavero I Gastaldi M , et al. HaNDL syndrome: a reversible cerebral vasoconstriction triggered by an infection? A case report and a case-based review. Eur J Med Res 2022; 27: 19. 36209134 10.1186/s40001-022-00815-8 PMC9548142

[22] Anderson D Nathoo N McCombe JA , et al. Anti-N-methyl-d-aspartate receptor encephalitis: a primer for acute care healthcare professionals. J Intensive Care Soc 2021; 22(2): 95–101. 34025748 10.1177/1751143720914181 PMC8120570

[23] Schwitter J Branca M Bicvic A , et al. Long-term sequelae after viral meningitis and meningoencephalitis are frequent, even in mildly affected patients, a prospective observational study. Front Neurol 2024; 15: 1411860. 39087005 10.3389/fneur.2024.1411860 PMC11288970

[24] Bodilsen J Dalager-Pedersen M Schønheyder HC , et al. Dexamethasone treatment and prognostic factors in community-acquired bacterial meningitis: a Danish retrospective population-based cohort study. Scand J Infect Dis 2014; 46: 418–425. 24645971 10.3109/00365548.2014.887223

[25] van de Beek D de Gans J Spanjaard L , et al. Clinical features and prognostic factors in adults with bacterial meningitis. N Engl J Med 2004; 351: 1849–1859. 15509818 10.1056/NEJMoa040845

[26] Dauchy FA Gruson D Chêne G , et al. Prognostic factors in adult community-acquired bacterial meningitis: a 4-year retrospective study. Eur J Clin Microbiol Infect Dis 2007; 26: 743–746. 17694339 10.1007/s10096-007-0381-6

[27] Domingo P Pomar V Benito N , et al. The changing pattern of bacterial meningitis in adult patients at a large tertiary university hospital in Barcelona, Spain (1982–2010). J Infect 2013; 66: 147–154. 23168216 10.1016/j.jinf.2012.10.030

[28] Sigurdardóttir B Björnsson OM Jónsdóttir KE , et al. Acute bacterial meningitis in adults: a 20-year overview. Arch Intern Med 1997; 157: 425–430. 9046894 10.1001/archinte.1997.00440250077009

[29] Venkatesan A Tunkel AR Bloch KC , et al. Case definitions, diagnostic algorithms, and priorities in encephalitis: consensus statement of the international encephalitis consortium. Clin Infect Dis 2013; 57(8): 1114–1128. 23861361 10.1093/cid/cit458 PMC3783060

[30] Iguchi M Noguchi Y Yamamoto S , et al. Diagnostic test accuracy of jolt accentuation for headache in acute meningitis in the emergency setting. Cochrane Database Syst Rev 2020; 2020(6): CD012824. https://pmc.ncbi.nlm.nih.gov/articles/PMC7386453/10.1002/14651858.CD012824.pub2 PMC7386453 32524581

[31] Contou D Painvin B Daubin D , et al. Hemodynamic and neurological presentations of invasive meningococcal disease in adults: a nationwide study across 100+ French ICUs : the RETRO-MENINGO study. Intensive Care Med 2025; 51: 1587–1602. 40794167 10.1007/s00134-025-08043-4

[32] Subbarao S Ribeiro S Campbell H , et al. Trends in laboratory-confirmed bacterial meningitis (2012-2019): national observational study, England. Lancet Regional Health 2023; 32: 100692. 10.1016/j.lanepe.2023.100692 PMC10393823 37538400

[33] Wilder-Smith A. Meningococcal vaccine in travelers. Curr Opin Infect Dis 2007; 20: 454–460. 17762777 10.1097/QCO.0b013e3282a64700

[34] Wikipedia. West Nile virus in the United States. Wikipedia (Online), https://en.wikipedia.org/w/index.php?title=West_Nile_virus_in_the_United_States&oldid=1292334251 (2025, accessed 11 September 2025).

[35] GOV.UK. First detection of West Nile virus in UK mosquitoes. GOV.UK (Online), https://www.gov.uk/government/news/first-detection-of-west-nile-virus-in-uk-mosquitoes (2025, accessed 11 September 2025).

[36] GOV.UK. Tick-borne encephalitis detection in England. GOV.UK, (Online), https://www.gov.uk/government/news/tick-borne-encephalitis-detection-in-england. (2023, accessed 11 September 2025).

[37] American Academy of Neurology. How to perform a lumbar puncture. American Academy of Neurology (Online), https://www.youtube.com/watch?v=a9LTezyBvbE (2025, accessed 11th September 2025).

[38] British Infection Association. Investigation of meningitis and encephalitis. British Infection Association (Online), https://www.britishinfection.org/application/files/1117/3615/0506/Investigation_of_Meningitis_and_Encephalitis.pdf (2024, accessed 11 September 2025).

[39] NICE. Meningitis (bacterial) and meningococcal disease: recognition, diagnosis and management. NICE Guideline 240. National Institute for Health and Care Excellence (NICE), 2024. 38843370

[40] Thakur KT Legouy C Sonneville R. Treating acute encephalitis. Intensive Care Med 2024; 50: 1916–1919. 39133286 10.1007/s00134-024-07569-3

[41] Hofmaenner DA Singer M. Challenging management dogma where evidence is non-existent, weak or outdated. Intensive Care Med 2022; 48: 548–558. 35303116 10.1007/s00134-022-06659-4 PMC8931587

[42] Martinez-Torres F Menon S Pritsch M , et al. Protocol for German trial of acyclovir and corticosteroids in Herpes-simplex-virus-encephalitis (GACHE): a multicenter, multinational, randomized, double-blind, placebo-controlled German, Austrian and Dutch trial [ISRCTN45122933]. BMC Neurol 2008; 8: 40. 18959773 10.1186/1471-2377-8-40 PMC2605746

[43] Meyding-Lamadé U Jacobi C Martinez-Torres F , et al. The German trial on Aciclovir and Corticosteroids in Herpes-simplex-virus-Encephalitis (GACHE): a multicenter, randomized, double-blind, placebo-controlled trial. Neurol Res Pract 2019; 1: 26. 33324892 10.1186/s42466-019-0031-3 PMC7650106

[44] Brophy GM Bell R Claassen J , et al. Guidelines for the evaluation and management of status epilepticus. Neurocrit Care 2012; 17: 3–23. 22528274 10.1007/s12028-012-9695-z

[45] Tetens MM Roed C Bodilsen J , et al. Use of intensive care, intracranial pressure monitoring, and external ventricular drainage devises in patients with bacterial meningitis, a cohort study. Acta Neurochir 2024; 166: 287. 38980542 10.1007/s00701-024-06188-7 PMC11233385

[46] Ajdukiewicz KM Cartwright KE Scarborough M , et al. Glycerol adjuvant therapy in adults with bacterial meningitis in a high HIV seroprevalence setting in Malawi: a double-blind, randomised controlled trial. Lancet Infect Dis 2011; 11: 293–300. 21334262 10.1016/S1473-3099(10)70317-0 PMC3999512

[47] Mourvillier B Tubach F van de Beek D , et al. Induced hypothermia in severe bacterial meningitis: a randomized clinical trial. JAMA 2013; 310: 2174–2183. 24105303 10.1001/jama.2013.280506

[48] Deliran SS Brouwer MC van de Beek D. Intracerebral haemorrhage in bacterial meningitis. J Infect 2022; 85: 301–305. 35728645 10.1016/j.jinf.2022.06.013

[49] McGill F Heyderman RS Panagiotou S , et al. Acute bacterial meningitis in adults. Lancet 2016; 388: 3036–3047. 27265346 10.1016/S0140-6736(16)30654-7

[50] Parents say daughter, 15, ‘let down by NHS’ after meningitis death [Internet]. BBC News (Online), https://www.bbc.com/news/articles/c4g5jy7yz81o (2025, accessed 11 September 2025).

